# Clinical Audit on Off-Label and Unlicensed Medication Prescribing Practices in the Stanley Treatment and Intervention Team, Derwent Clinic

**DOI:** 10.1192/bjo.2026.11750

**Published:** 2026-06-30

**Authors:** Ernesto Vera, Ahmed Abbas, Ahmed Mostafa, Eman Arebi

**Affiliations:** Derwent Clinic, Consett, United Kingdom

## Abstract

**Aims:**

- To monitor adherence to the General Medical Council professional standards in prescribing unlicensed medications for patients open to the Stanley Treatment and Intervention team.
- To identify areas of unlicensed prescribing that require improvement in order to communicate this with prescribers to raise awareness of the professional standards and reinforce them.
- To ensure good practice in decision-making around treatment choice in conjunction with parents and carers
- To allow use of unlicensed or off-label medicines in line with recognised clinical practice and/or published evidence
- To minimise risk associated with unlicensed and off-label use of medicines.

**Methods:**

A search was conducted on clinic letters by medical staff in the Stanley Treatment and Intervention Team in community mental health between 1 July 2024 and 31 October 2024. We randomly chose 27 clinic letters.

To complete this audit, we reviewed all 27 patient’s electronic record to identify unlicensed medication use practice using the audit tool.

**Results:**

The total number of patients included in this audit was 27.The number of patients who were initiated on unlicensed medications was 2 out of 27 . Out of these, 2 patients records had clear documentation of discussion of the use of unlicensed medication with the patient, 2 patients records had a clear documentation of the rationale for prescribing a medicine off-label or prescribing an unlicensed medicine and 2 patients records had a clear documentation of the benefit vs risk of unlicensed medication use.

Subsequently, 2 patients records had documentation of clear, accurate and legible record of all medicines prescribed, 2 patients records had clear documentation that sufficient information about the medicines were given to patient, or their parents or carers. Lastly, there were no patients considered to be in a vulnerable group such as children and adolescents, women of childbearing age and elderly patients.

**Conclusion:**

The Stanley Treatment and Intervention Team in community mental health was found to be 100% compliant with the General Medical Council standards. Compared to a previous audit in the inpatient ward, we identified this was due to improved documentation, open discussions with patients and clearly documented rationale. We suggest practices to have a set proforma to support doctors identifying a rationale, prescribing and documenting commencement of unlicensed medications.

